# Editorial: Physiological, molecular and genetic mechanisms of abiotic stress tolerance in tropical crops

**DOI:** 10.3389/fpls.2023.1150343

**Published:** 2023-02-10

**Authors:** Zhijian Chen, Jacobo Arango, Idupulapati M. Rao, Cuiyue Liang

**Affiliations:** ^1^ Institute of Tropical Crop Genetic Resources, Chinese Academy of Tropical Agricultural Sciences, Haikou, China; ^2^ International Center for Tropical Agriculture, Cali, Colombia; ^3^ International Centre of Insect Physiology and Ecology(icipe), Nairobi, Kenya; ^4^ Root Biology Center, State Key Laboratory for Conservation and Utilization of Subtropical Agro-bioresources, College of Natural Resources and Environment, South China Agricultural University, Guangzhou, China

**Keywords:** drought, chilling, low phosphorus, gene expression, multi-omics

## Introduction

The tropics are either the center of origin or domestication of many of the economically important crops currently cultivated in the world (Phalan et al., 2013; Fern and Fern, 2014; Laurance et al., 2014). Abiotic stresses, such as drought, chilling, and nutrient deficiency, are the major constraints for plant growth and productivity (Zhu, 2016). Therefore, an improved understanding of the physiological, biochemical, and molecular responses and tolerance mechanisms, along with the discovery of novel stress-responsive pathways and genes, may contribute to efficient breeding strategies that could improve abiotic stress tolerance in tropical crops.

Tropical crops have evolved a number of complex mechanisms for abiotic stress tolerances. These mechanisms contribute to greater physiological performance through stress perception, signal transduction, transcriptional activation of stress-responsive target genes, and synthesis of stress-related proteins, metabolites and other molecules (Pardo and VanBuren, 2021). It is therefore imperative to accelerate the research efforts to unravel the mechanisms underlying abiotic stress tolerance in tropical crops. The research topic contains seven articles that addressed mechanistic aspects of crop tolerances to different abiotic stress factors in the tropics.

## Tolerance to drought and chilling

The article of Fonta et al. provided insights into root phenotypes in response to drought stress in rice (*Oryza sativa*) and identified several potential root phenotypes that resulted in improved relative performance under drought stresses. These integrated phenotypes could serve as selection targets in ideotype breeding for improved drought tolerance in rice. In another study, Xu et al. dissected the function of PIN-FORMED auxin efflux carrier (*OsPIN9*) in modulating chilling tolerance in rice. *ospin9* mutants were generated and these displayed tolerance to chilling stress, which was mainly attributed to effective scavenging of reactive oxygen species (ROS). This knowledge will contribute to improved understanding of the *OsPIN9* gene in regulating chilling tolerance by modulating ROS homeostasis in rice.

## Tolerance to nutrient stress

Wu et al. determined the effects of phosphorus (P) fertilizer on soil P forms, bacterial diversity and community composition in the rhizosphere soils of sugarcane (*Saccharum officinarum* L.). Long-term P fertilization significantly changed the bacterial structure and functions, which may be beneficial for soil P cycling. This study provides useful information for improving soil P availability through the interaction between the microbes and P fertilization. Ou et al. reported a phosphate (Pi) starvation-responsive NUCLEAR FACTOR Y subunit alpha gene, *GmNF-YA8*, in soybean (*Glycinme max*). Overexpression of *GmNF-YA8* inhibited plant growth and delayed flowering, but stimulated the emergence of lateral root primordium under low P conditions. The study provides insights into the roles of *GmNF-YA8* in regulating plant growth and response to nutrient stress.

## Postharvest physiological deterioration

Cassava (*Manihot esculenta* Crantz) is an important tropical crop in the world. An et al. investigated the potential role of basic helix-loop-helix (*bHLH*) gene family in cassava. Several *MebHLH* genes were proposed to be involved in the regulation of postharvest physiological deterioration (PPD) process. Silencing of *MebHLH72* and *MebHLH114* led to a decreased linamarin content in leaf of cassava. This study provided improved understanding of the functions of *MebHLHs* in PPD tolerance and cyanogenic glucosides biosynthesis in cassava.

## Seed shattering

Stylo (*Stylosanthes* spp.) is an important tropical forage. Stylo accessions with shorter lobules and thicker stems had a lower seed shattering rate and a higher seed weight as reported by Li et al. These authors found that the tearing of the abscission zone occurred probably due to the intense enzymatic degradation of polygalacturonase and cellulase. Furthermore, genes related to lignin biosynthesis, cellulase synthesis, and plant hormone signal transduction were highlighted and might function in stylo seed shattering.

## Gene expression and stress resistance

Seashore paspalum (*Paspalum vaginatum* Swartz) is an important halophytic perennial grass with multiple uses in tropical areas. Hao et al. investigated the N6-methyladenine (6mA) DNA modification in the *P. vaginatum* genome. 6mA modification sites were found to be broadly distributed across the *P. vaginatum* genome, and the moderate 6mA density genes were correlated with stress resistance. This study provides new insights into the association between 6mA methylation and gene expression in a perennial grass.

## Conclusion and future perspective

This Research Topic provides important updates on understanding the mechanisms of adaptation of tropicl crops to abiotic stresses, and highlights some of the important factors contributing to abiotic stress tolerance, which are essential for genetic improvement of tropical crop varieties with high yield and nutritional quality but with less inputs under stress conditions. Since tropical crops are considered as economically important dietary sources for human health and nutrition, this Research Topic also emphasizes the pressing need for improving the performance of tropical crops for the global population.

Although significant progress has been made in understanding abiotic stress tolerance in tropical crops, the specific mechanisms need to be further defined. Thus, future research efforts should focus on (i) investigation of integrated phenotypic traits of tropical crops for improving crop yield across diverse stress environments; (ii) making full use of the multi-omics (e.g., phenomics and genomics) analysis for accelerating genetic improvement of tropical crops that are able to cope with abiotic stress factors; (iii) application of quantitative trait loci (QTL) and genome-wide association study (GWAS) of agronomic traits linked with stress tolerance to identify key traits and the genes associated with specific traits.

## Author contributions

All authors listed have made a substantial, direct, and intellectual contribution to the work and approved it for publication.
